# Miscellaneous Items

**Published:** 1856-01

**Authors:** 


### Miscellaneous Items.

On the Healing of Abscesses by the First Intention. By M. Chaissaignac.—M. Chaissaignac has for some time past endeavored to unite abscesses by the first intention, after their complete evacuation; and he reports that his success has been very considerable. His method of procedure may be judged of by the narration of a case which recently occurred at the Lariboisere Hospital, and was observed by all who attend there. A healthy man, aged nineteen, was admitted February 17th, presenting all the symptoms of acute abscess of the axilla, which had been about a week in forming. On the 19th, chloroform having been given, a considerable quantity of well-conditioned pus was discharged by the bistoury, pressure being exercised in all directions for the purpose of securing complete evacuation. The cavity of the abscess was next thoroughly washed out with water introduced through the tube of an irrigator, in order to bring away any remaining pus, the injection being continued until the water returned completely limpid. Pressure was again employed to force out every drop of the water, and the orifice was strapped up. A large pad of charpie was introduced into the axilla in order to make pressure over that region, and the arm was confined in one of Mayor's bandages, as if for fracture of the clavicle. On the 21st, cicatrization was complete, no discharge of the pus whatever being visible. The bandage was continued as a matter of precaution for two or three days, and then the arm was allowed to hang down, no pain being produced. In the site of the abscess a little indurated spot could be felt. On the 27th he was discharged, quite well.—*B. & F. Med. Chir. Rev., from Gazette des Hopitaux*, 1855, *Fo.* 38.

Physicians.—The editor of the *American Medical Gazette* estimates that one physician is required for every 700 inhabitants. This would give over 30,000 in the nation. It is probable that the average life of a physician is not over thirty years, after he enters his profession. Hence it follows by computation that the waste, by deaths alone, is one thousand physicians a year. But more are lost by change of business than by death; and this loss might be estimated, without exaggeration, at 300. We have, then, an actual loss of 1300 physicians annually, which must be made up. Again, the increase of population in the United States is about 700,000 a year, which it requires an increase of 1,000 physicians to supply. There are, therefore, actually required *twenty-three hundred* new physicians in the United States yearly. To meet this, we have only 1,400 graduates from the schools, and about 300 foreigners, making in all 1,700, and leaving an actual deficiency of 600 a year, to be supplied by irregular practitioners.

American Physicians in the Russian Service.—The following is a translation of a contract made by an American physician and the agent of the Czar: "Mr. S——, physician, will receive as compensation the sum of sixty silver roubles (not quite fifty dollars), which will be paid in current sounding silver, in the course of the first three days of every month. Besides, there will be given to Mr. S——, physician, enough money to pay for a first class passage from Berlin to Warsaw. At Warsaw, the imperial Russian Government will provide him with the means to conduct him to his place of destination. The expenses of his return to Berlin, in case he shall cancel this contract, will be provided by the imperial government, as well as official voyages, which will be paid for according to the rules. The time of service will be counted as commencing the day that Mr. S——, physician, shall depart from here to Warsaw."—*London Lancet.*

Aneurism of the Superior Palatine Artery. By M. Thierlinck.—This surgical curiosity was met with in the case af a man, æt. 74. The tumor occupied the roof of the palate, which bled so frequently that the patient was much exhausted. The tumor was soft, elastic, and pulsated synchronously with the heart, alternately expanding and diminishing. Its cause was unknown, and it had lasted for three weeks. The actual cautery was employed, the slough separated in eight days, the hemorrhage did not recur, and a perfect cure resulted.—*Dublin Hospital Gaz.*, from *Gazette Medicale.*

American Surgeons in the Russian Army.—There are eight American Surgeons attached to the Russian army in the Crimea: Drs. Eldridge, Jones, Johnson and Stoddard, from Maryland; Drs. Reed and Denninger, from Pennsylvania; Dr. Holt, from S. Carolina; Dr. Smith, from Louisiana; who are all treated with much consideration and respect by the Russian officers.

Antidote to Strychnia.—Extract of a letter from Wm. Nick Pindell, M.D., of St. Michael's, Talbot county, Maryland, to the editor. The object of this letter is merely to state facts, and through the Journal to have the facts made known, at the same time to obtain through your influence further experiments, either to satisfy or dispel from the minds of some that there is always at hand a "safe" and "sufficient" antidote to the poisonous "strychnia." Will you have the "antidote" fully tested?

I will now state the experiments of mine own, and the occasion of their having been made.

There were some three or four dogs, nightly frequenting my premises, committing devastation upon anything left exposed. They had continued to worry me until forbearance ceased to be a virtue, and sentence was pronounced: "They shall die." A piece of meat, containing "1 grain of strychnia," was placed close beside a jar of refuse "lard." I sat, watched a dog take, eat the meat, and commence upon the lard. My watch was beside me, and I expected the dog to die; five, ten, fifteen, twenty, thirty minutes passed; still, he did not die. That night, the "lard" having all been eaten, three pieces of meat, upon which the poison had been placed, were dropped in separate places. Next morning I found three dogs dead. I, that day, tried dog No. 1 with two more grains, without the lard, and in ten minutes, he was dead. There have been nine instances in which the poison was given, and the antidote used; in neither one did the dog die. In eleven, without the lard, they all died. The half grain was sufficient to kill. Three grains failed when the antidote was used.

The test has also been used upon cats with the same result. A difference in time of death is made by simply putting it upon *fat* instead of *lean* meat; the latter being over some three minutes sooner than the former.

It is proper to state, that the lard was given in not less than "half-pints" up to the one and a-half.

What is the action of the lard?

Will you, doctor, have this matter tested fully, and then report through the Journal? The article of strychnia, which I have used, was obtained from my chemists in Baltimore, Coleman & Rogers. I do not know, or am unable to account for the action of the lard. I may now state that I have used camphor, and "failed."

If it be necessary, it will afford me pleasure to report at length of the trials, and then have the public to know of the agent, if it should meet your wishes.

St. Michael's, Talbot Co., Md., July 12, '55,

Atmospheric Pressure Plates.—In the April number of the Dental Register, Dr. John Harris asks several questions in regard to air chambers with plates, &c.

We do not intend to attempt to answer these questions, for the simple reason that we cannot, and like Dr. Harris, we are anxious for a solution of the same questions, and we confess to the same state of uncertainty in regard to the establishing of a principle that shall tell us when to adopt the central cavity with full plate, or the ridge cavity with narrow plate, and when to use a plain plate with no air chamber at all.

We find ourself adopting all these styles after a careful study of the different cases presented. We do not design to go into the discussion of the subject at present, for we lack really decided opinions on the subject. Our object at present is, to call attention to one fact that we are sure of, which is, that a full plate extending over the alveolar ridge far enough to fill out the muscles of the face to near their normal tension, and to offer the contact of a round edge to them, will never fail of a good "suction plate," if fitted accurately with or without the central cavity.—*Dental Obturator.*

A Restless Tongue.—A Boston lady has at this time a somewhat novel disease: a continual motion of the tongue, which no device, effort of the will, or medication, controls. We do not mean that she is a nuisance as a talker or a retailer of street gossip. On the contrary, a worthier woman does not exist. She has expended five hundred dollars among the dentists for artificial teeth, which her unruly member has knocked out so repeatedly, that they are now wholly abandoned; she is therefore a good representative of one of Shakspeare's seven ages—viz. *sans teeth.* Her tongue is moving involuntarily within the mouth, and against the walls of the cheeks. In conversation, the organ takes on a normal action, but runs into its usual rapidity of motion at the conclusion of a sentence.

Longevity of the Negro.—A letter from Rio Janeiro, in the *Corrierre Mercantile* of Genoa, mentions a slave 109 years old, of the name of Francesco Tomassa Da Sala, now living on a plantation a few miles from the capital. He was born in 1747, and had fourteen sons, who became fathers of 160 grandchildren, from whom sprung 70 great grandchildren, having in their turn, up to the present time, produced five childreen, making a grand total of 249 persons, issued from one stock, still alive.—*Virginia Med. and Sur. Jour.*

